# Editorial: Neural Dynamics – Models and Complexity

**DOI:** 10.3389/fnins.2021.841077

**Published:** 2022-05-06

**Authors:** Paul A. Watters, Plamen Ch. Ivanov, Xinbao Ning, Wei Wang

**Affiliations:** ^1^Cyberstronomy Pty Ltd., Sydney, NSW, Australia; ^2^Boston University, Boston, MA, United States; ^3^Department of Electronic Science and Engineering, Nanjing University, Nanjing, China; ^4^Department of Educational Technology, Nanjing Normal University, Nanjing, China

**Keywords:** EEG, complexity, chaos, brain dynamics, Brain-Computer Interface (BCI)

The fundamental mysteries of the brain remain fundamentally uncertain, despite numerous attempts at quantification, measurement, modeling, explanation, and prediction. The papers presented in this special issue attempt to progress the field according to the time-honored traditions of academic exegeses undertaken within a scientific framework—there are new models, new experiments, and attempts to link the two. Fundamental progress using the scientific method is—by its very—slow but exhaustive, innovative yet rigorous, broad but also very deep. When we integrate disparate results together that provide predictable yet also explanatory theories which are not falsifiable, we may look toward further progress in providing satisfying and valid explanations of the phenomena which make humankind apparently unique. These include all aspects of cognitive and social development, but broader and unique characteristics, such as creativity and altruism. These phenomena are the hardest to explain using “data mining” approaches which can provide model predictability, but often lack explanatory mechanisms.

When we talk about brain dynamics, we are really looking for explanations as to how observable physiological activity characterizes and predicts the spectrum of human behavior, including emotion classification, detection of epileptic seizures, Alzheimer's and Parkinson's Disease identification, mental workload assessment, developmental disorder assessment, controlling the Brain-Computer Interface (BCI), the effects of physical activity and exercise on brain dynamics and cognitive function, and the sleep-wake cycle under sleep disorders. The papers in this special issue speak closely to many of these themes, but also invite further, deeper questions about criticality and self-organization in neuronal populations and brain dynamics, brain dynamics and neuronal interactions, and the observable measures of complexity that can be reliably extracted from noisy signals.

We commend the work to you—the reader—who is seeking deeper answers than what can typically be obtained through application of the “linear systems” view of the world.

## Author Contributions

All authors listed have made a substantial, direct, and intellectual contribution to the work and approved it for publication.

## Conflict of Interest

PW is employed by the company Cyberstronomy Pty Ltd. The remaining authors declare that the research was conducted in the absence of any commercial or financial relationships that could be construed as a potential conflict of interest.

## Publisher's Note

All claims expressed in this article are solely those of the authors and do not necessarily represent those of their affiliated organizations, or those of the publisher, the editors and the reviewers. Any product that may be evaluated in this article, or claim that may be made by its manufacturer, is not guaranteed or endorsed by the publisher.

